# RNA Biomarkers in Bipolar Disorder and Response to Mood Stabilizers

**DOI:** 10.3390/ijms241210067

**Published:** 2023-06-13

**Authors:** Claudia Pisanu, Alessio Squassina

**Affiliations:** 1 Department of Biomedical Sciences, Section of Neuroscience and Clinical Pharmacology, University of Cagliari, 09042 Monserrato, Italy; claudia.pisanu@unica.it; 2 Department of Psychiatry, Faculty of Medicine, Dalhousie University, Halifax, NS B3H 2E2, Canada

**Keywords:** bipolar disorder, biomarker, transcript, microRNA, miRNA, circular RNA, circRNA, lithium, pharmacogenetics, precision medicine, personalized medicine

## Abstract

Bipolar disorder (BD) is a severe chronic disorder that represents one of the main causes of disability among young people. To date, no reliable biomarkers are available to inform the diagnosis of BD or clinical response to pharmacological treatment. Studies focused on coding and noncoding transcripts may provide information complementary to genome-wide association studies, allowing to correlate the dynamic evolution of different types of RNAs based on specific cell types and developmental stage with disease development or clinical course. In this narrative review, we summarize findings from human studies that evaluated the potential utility of messenger RNAs and noncoding transcripts, such as microRNAs, circular RNAs and long noncoding RNAs, as peripheral markers of BD and/or response to lithium and other mood stabilizers. The majority of available studies investigated specific targets or pathways, with large heterogeneity in the included type of cells or biofluids. However, a growing number of studies are using hypothesis-free designs, with some studies also integrating data on coding and noncoding RNAs measured in the same participants. Finally, studies conducted in neurons derived from induced-pluripotent stem cells or in brain organoids provide promising preliminary findings supporting the power and utility of these cellular models to investigate the molecular determinants of BD and clinical response.

## 1. Introduction

Bipolar disorder (BD) is a severe chronic psychiatric disorder characterized by episodes of mania or hypomania, alternating with depression. Because of its early onset, prevalence of more than 1% of the global population, high rate of psychiatric and medical comorbidities and increased premature mortality, BD represents one of the main causes of disability among young people [1]. Both genetic and environmental factors are known to contribute to the onset of BD, and heritability of this disorder has been estimated at 60–85%. Genome-wide association studies (GWAS) have successfully identified a number of genetic loci implicated in this disorder [2,3]. However, the causes of BD, as well as the biological networks involved in this disorder, are still largely unknown. In addition, an accurate and timely diagnosis is difficult, as no biomarker is available and the clinical presentation of BD is often a depressive episode similar to unipolar depression [1]. 

Pharmacological treatment represents the mainstay in the long-term management of BD. Among mood stabilizers, lithium represents a first-line option because of its effectiveness in the acute phase of the disorder, in the prevention of recurrences and in the reduction of suicide risk. However, clinical response to lithium presents a high interindividual variability, with approximately 70% of patients showing partial or nonresponse [4]. Lithium response is heritable, and initiatives such as the International Consortium on Lithium Genetics (ConLiGen) are contributing to knowledge on the molecular determinants underlying this trait [5]. The first GWAS conducted by ConLiGen suggested the involvement of long noncoding RNAs (lncRNAs) in lithium response [5], and subsequent secondary analyses pointed to different markers and pathways potentially playing a role in this trait [6,7,8]. However, the molecular players underlying lithium’s complex mechanism of action are still elusive, and no reliable biomarkers are available to identify patients who might respond to this drug [9]. Even less information is available regarding molecular markers involved in the response to other mood stabilizers, such as the anticonvulsants carbamazepine, valproate and lamotrigine. The identification of reliable biomarkers that respond to mood stabilizers is a priority, since demonstration of proven clinical efficacy is one of the most important factors required for the successful implementation of genomic medicine in the health-care system, together with cost-effectiveness, appropriate knowledge and education and appropriate policy and legislation [10].

Transcriptomic studies provide information complementary to GWAS, allowing for the study of the dynamic evolution of different types of RNA markers based on specific tissues or biofluids, cell types and developmental stage. Among the most investigated RNA biomarkers in BD and in response to mood stabilizers, there are messenger RNA (mRNA) and different types of noncoding RNAs, such as microRNAs (miRNAs), circular RNAs (circRNAs) and lncRNAs.

miRNAs are single-stranded RNA molecules, approximately 20–22 nucleotides in length, that play a substantial role in the regulation of gene expression. Their main mechanism of action consists in promoting gene silencing by guiding argonaute proteins to the 3’ region of a target mRNA, thus allowing for the recruitment of factors that promote translational repression or mRNA decay [11]. Because the majority of protein-coding genes have been shown to present at least one miRNA-binding site, miRNAs are involved in a wide range of cellular functions and biological processes, and their dysregulation has been linked to several human diseases, including psychiatric disorders [11,12]

CircRNAs are single-stranded RNA molecules produced by pre-mRNAs through a process called back-splicing [13]. Unlike linear RNAs, circRNAs are characterized by a covalently closed-loop structure. This conformation makes circRNAs resistant to degradation by exonucleases and more stable compared with other transcripts. CircRNAs were originally considered a result of “splicing noise” with no relevant biological significance. However, in the last few years it has been shown that circRNA molecules are conserved, tissue-specific and involved in relevant biological functions [14]. While their roles are still largely elusive, circRNAs have been suggested to be involved in the regulation of transcription, protein transport and protein–protein interactions. In particular, some circRNAs present miRNA-binding sites and may act as miRNA sponges, sequestering miRNAs and, thus, preventing their interaction with mRNA targets. The prevalence of circRNAs has been largely underestimated until recently because their identification presents technical challenges. In fact, microarrays do not allow the detection of circRNAs or to distinguish the expression of circRNAs from their linear host genes. In addition, since most early RNA sequencing studies focused on measuring levels of RNA with polyA tails, they involved a polyA RNA enrichment step that led to the depletion of circRNAs. Modern RNA sequencing and bioinformatic pipelines are able to identify these markers, which allowed for the realization that circRNAs are much more abundant then first hypothesized. Most circRNAs show specific expression patterns based on tissue, cell type and/or developmental stage and, intriguingly, circRNAs are enriched in brain tissues compared with other tissues [14]. Specifically, in the brain, a significantly greater number of genes, particularly synaptic genes, host circRNAs [14]. In addition, their stability and low turnover rate suggest they might accumulate in postmitotic cells, such as neurons [14]. The abundance of circRNAs in the brain, as well as the peculiar characteristics of these markers, contributes to the increased research interest on these transcripts as potential biomarkers of brain disorders.

LncRNAs are a family of noncoding RNA molecules longer than 200 nucleotides and characterized by substantial differences in terms of length, expression patterns and longevity. Several lncRNAs have been shown to be able to regulate the expression of nearby or distant genes. In addition, lncRNAs can modulate chromatin structure, response to DNA damage and different signaling pathways [15]. As in the case of circRNAs, the measurement of lncRNAs is associated with specific technical challenges because of their generally low abundance. However, while the number of studies exploring these markers is still limited compared with other transcripts, over the last few years there has been increased interest in lncRNAs as disease biomarkers and potential therapeutic targets due to their high specificity in tissue expression patterns, fast turnover and regulation of cellular networks [15].

In this narrative review on human studies, we describe the most promising findings regarding the potential utility of transcripts as diagnostic markers of BD, as well as markers of response to mood stabilizers. To this aim, in Section 2 we describe studies that measured levels of mRNAs or noncoding RNAs in biofluids, peripheral cells or cellular models derived from patients with BD compared with healthy controls (HCs) or with patients with other psychiatric disorders, such as major depressive disorder (MDD) or schizophrenia (SZ). Next, in Section 3, we revise studies that analyzed the potential role of transcripts as biomarkers of response to mood stabilizers in patients with BD.

## 2. RNA Biomarkers and Bipolar Disorder

In this section, we present studies investigating the association of RNAs with BD diagnosis or course. We present, separately, studies investigating peripheral levels of RNA markers in biofluids or peripheral cells derived from patients with BD (Section 2.1) or cellular models derived from patients with BD (Section 2.2), including lymphoblastoid cell lines (LCLs), induced pluripotent stem cells (iPSCs), iPSC-derived neural precursors cells (NPCs) or iPSC-derived neurons or brain organoids. Studies that conducted measurements fitting in more than one of these categories are described in the one deemed to be the most relevant based on the reported findings.

### 2.1. Peripheral Levels of RNA Markers in Biofluids or Peripheral Cells from Patients with BD

Studies investigating the association between RNA markers and BD in biofluids or peripheral cells are presented in Table 1. The majority of the studies we retrieved investigated only one type of RNA (mRNAs or a type of noncoding RNA), while only few studies conducted integrated analyses of mRNAs and either miRNAs [16], circRNAs [17] or lncRNAs [18,19]. All of the studies that conducted integrative analyses used a genome-wide approach, except the study from Eghtedarian and colleagues, who measured levels of different mRNAs and lncRNAs related to a specific pathway (the vitamin D receptor pathway) [18]. While including a relatively limited number of participants (a discovery cohort of 4 patients with BD and HCs and a validation cohort of 16 patients with BD and HCs), the study from Fu and colleagues presented a particularly interesting integrative approach [17]. Namely, the authors measured genome-wide levels of mRNAs and circRNAs, predicted miRNA targets of the top 10 upregulated and the top 10 downregulated circRNAs and then constructed circRNA–miRNA–mRNA networks altered in patients with BD compared with HCs [17]. Functional enrichment analysis suggested differentially expressed mRNAs to be involved in processes such as regulation of cell growth, immune imbalance and inflammatory response [17].

The large majority of the studies presented in Table 1 were conducted in whole blood or peripheral blood mononuclear cells (PBMCs), with only a minority of studies exploring RNA levels in plasma/serum [16,20,21,22,23] or plasma-derived extracellular vesicles (EVs) [24,25]. Among EVs, exosomes are membrane vesicles released by different cells into the extracellular matrix that play a pivotal role in intercellular communication and signal transmission through the transfer of bioactive molecules to adjacent or distant recipient cells [26]. Exosomes carry a variety of molecules, including metabolites, lipids and nucleic acids, and are enriched in miRNAs. Intriguingly, neural exosomes can cross the blood–brain barrier and can be detected peripherally. Therefore, the change of peripheral exosomal content in patients with BD might, at least, partly reflect central changes, thus potentially allowing to identify brain-relevant biosignatures of disease and drug response in a noninvasive way. The few available studies that explored miRNA levels in EVs and exosomes reported promising results. Ceylan and colleagues measured genome-wide levels of miRNAs in plasma exosomes from 69 patients with BD (15 depressed, 27 manic and 27 euthymic) and 41 HCs. After multiple testing correction, three miRNAs showed lower levels (miR-484, miR-652-3p and miR-142-3p) and one miRNA higher level (miR-185-5p) in patients with BD compared with HCs [25]. The predicted targets of the four miRNAs were enriched for different pathways, including PI3K/Akt signaling, fatty acid biosynthesis/metabolism, extracellular matrix and adhesion pathways. No miRNA was significantly altered among the different states of BD [25]. Conversely, other studies suggested the potential utility of miRNAs as disease state markers. Namely, Camkurt and colleagues measured the levels of eight candidate miRNAs (selected based on previous evidence of their potential involvement in psychiatric disorders) in whole blood from 58 patients with BD (19 manic and 39 euthymic) and 51 HCs [27]. The levels of miR-07 were found to be significantly higher in patients with BD compared with HCs but also in patients in a manic episode compared with euthymic patients. Another study conducted by Banach and colleagues observed the downregulation of three miRNAs (miR-499, miR-798 and miR-1908) in patients with BD during a depression episode compared with a euthymic state [28].

Some studies identified significant differences in the RNA levels based on BD subtype. D’Addario and colleagues measured the mRNA levels of six candidate genes interacting with the brain-derived neurotrophic factor (BDNF) in PBMCs from 54 patients with BD type 1 (BD I), 45 with BD type 2 (BD II) and 42 controls. The authors reported lower levels of the prodynorphin (PDYN) gene in patients with BD II but not BD I compared with HCs. In addition, this study observed increased methylation at the *PDYN* promoter, as well as higher levels of genes involved in methylation, such as DNA methyltransferase 3 beta (DNMT3b) and methyl-CpG-binding protein 2 (MECP2) in patients with BD II compared with HCs. Other studies included a sample of patients with different psychiatric disorders, such as MDD [16,29] or SZ [30], aiming to distinguish among RNA markers specifically associated with BD or shared among different psychiatric disorders. Among these studies, Maffioletti and colleagues measured genome-wide miRNA levels in whole blood from 20 patients with BD, 20 with MDD and 20 HCs [29]. The study reported levels of five miRNAs to be increased specifically in patients with BD compared with HCs (hsa-miR-140-3p, hsa-miR-30d-5p, hsa-miR-330-5p, hsa-miR-378a-5p and hsa-miR-21-3p), while hsa-miR-330-3p and hsa-miR-345-5p showed higher levels in patients with either BD or MDD. However, one the miRNAs specifically associated with BD was found to be altered in MDD patients after treatment with antidepressants in a previous study conducted by the same authors [31]. Another study aimed at identifying the biosignatures of bipolar from unipolar depression measured genome-wide plasma miRNA levels in a discovery cohort of seven patients with BD, seven with MDD and six HCs [20]. The study reported higher levels of miR-19b-3p in patients with BD compared with patients with MDD, a result that was validated in a cohort of 27 patients with BD and 32 with MDD. In silico analyses suggested this miRNA to be involved in inflammatory dysregulation associated with experiencing early childhood trauma [20]. As shown in Table 1, a number of studies provided evidence of a good performance of the investigated RNAs in the discrimination of patients with BD from HCs, based, for example, on the area under the curve (AUC) [32]. However, it must be considered that several of these studies did not include a replication cohort.

**Table 1:** Studies evaluating the association between RNA markers and bipolar disorder in biofluids or peripheral cells.

Ref.	Sample	RNA Source	RNA Type	Method	Targets	Main Findings
[28]	15 patients with BD and 17 with MDD during a depression episode and at remission	Whole blood	miRNA	qPCR	miR-499, miR-798 and miR-1908	Lower levels of all miRNAs in the depressive state compared with remission exclusively in patients with BD
[27]	58 patients with BD I (19 manic and 39 euthymic) and 51 HCs	Whole blood	miRNA	qPCR	miR-26b-5p, miR-9-5p, miR-29a-3p, miR-106a-5p, miR-106b-5p, miR-107, miR-125a-3p and miR-125b-5p	Levels of miR-29a-3p, miR-106b-5p, miR-107 and miR-125a-3p were significantly higher in patients with BD compared with HCs. Levels of miR-106a-5p and miR-107 were significantly higher in manic patients compared to euthymic patients
[25]	69 patients with BD (15 depressed, 27 manic and 27 euthymic) and 41 HCs	Plasma exosomes	miRNA	qPCR	Genome wide	Levels of miR-484, miR-652-3p and miR-142-3p were lower, while levels of miR-185-5p were higher in patients with BD compared with HCs. No alterations among different states of BD
[20]	Discovery cohort: 7 patients with BD, 7 with MDD and 6 HCs; validation cohort: 27 patients with BD and 32 with MDD	Plasma	miRNA	Microarray, qPCR	Genome wide	Higher levels of miR-19b-3p in patients with BD compared with patients with MDD
[33]	54 patients with BD I, 45 with BD II and 42 HCs	PBMC	mRNA	qPCR	*COMT*, *DNMT*, *GAD67*, *MECP2*, *PDYN* and *SERT*	Lower *PDYN* expression in patients with BD II but not BD I compared with HCs. Higher levels of genes involved in methylation, such as *DNMT3b* and *MECP2*, in patients with BD II compared with HCs
[34]	169 patients with BD and 211 HCs	Whole blood	mRNA	qPCR	*NOTCH4*	Higher *NOTCH4* levels in patients with BD compared with HC
[18]	50 patients with BD I and 50 HCs	Whole blood	mRNA, lncRNA	qPCR	*SNHG6*, *MALAT1*, Linc00511, Linc00346, *VDR* and *CYP27B1*	Levels of *VDR*, *SNHG6*, *CYP27B1*, *MALAT1* and Linc00346 were higher in patients compared with HCs
[24]	20 patients with BD I and 21 HCs	Plasma EV	miRNA	Microarray	Genome wide	33 nominally significant microRNAs altered in patients with BD
[17]	Discovery cohort: 4 patients with BD and 4 HCs; validation cohort: 16 patients with BD and 16 controls	Whole blood	mRNA, circRNA	NGS and qPCR	Genome wide	50 circRNAs and 244 mRNAs showed nominally significant higher levels, while 44 circRNAs and 294 mRNAs lower levels in patients with BD compared with HCs
[16]	Discovery cohort: 18 patients with BD, 44 with MDD, and 31 HCs; validation cohort: 26 patients with BD, 84 with MDD and 74 HCs	Plasma	mRNA, miRNA	NGS and qPCR	Genome wide	Higher levels of hsa-let-7e-5p and hsa-miR-125a-5p in patients with either BD or MDD compared with HCs
[19]	Discovery cohort: 4 patients with BD and 4 HCs; validation cohort: 130 patients with BD and 116 HCs	Whole blood	mRNA, lncRNA	NGS and qPCR	Genome wide	Higher levels of the NR_028138.1 lncRNA in patients with BD compared with HCs
[21]	Discovery cohort: 3 patients with BD II and 3 HCs; validation cohort: 99 patients with BD II and 115 HCs	Serum	miRNA	NGS and qPCR	Genome wide	Levels of miR-7-5p, miR-23b-3p, miR-142-3p, miR-221-5p and miR-370-3p were higher in patients with BD II compared with HCs
[32]	51 patients with BD and 116 HCs	White blood	mRNA	qPCR	*COMT*, *GCAT*, *NRG1*, *PSAT1*, *SHMT2*, *SLC1A4* and *SRR*	A logistic ridge regression model including age, gender and mRNA expression levels of the 7 *NMDAR* genes showed an AUC of 0.92 in differentiating patients with BD from HCs
[29]	20 patients with BD, 20 with MDD and 20 HCs	Whole blood	miRNA	Microarray and qPCR	Genome wide	5 miRNAs showed higher levels specifically in patients with BD compared with HCs (hsa-miR-140-3p, hsa-miR-30d-5p, hsa-miR-330-5p, hsa-miR-378a-5p and hsa-miR-21-3p), while hsa-miR-330-3p and hsa-miR-345-5p showed higher levels in both BD and MDD
[30]	19 patients with BD, 20 with SZ and 20 HCs	PBMC	circRNA	NGS and qPCR	Genome wide	Levels of 30 circRNAs were specifically altered in patients with BD compared with HCs
[35]	50 patients with BD and 50 HCs	PBMC	lncRNA	qPCR	3 lncRNAs related to oxidative stress (lincRNA-p21, lincRNA-ROR and lincRNA-PINT)	Levels of all lncRNAs were lower in patients with BD, and specifically in male patients with BD, compared with HCs
[36]	50 patients with BD and 50 HCs	PBMC	lncRNA	qPCR	5 lncRNAs related to oxidative stress (*H19*, *LUCAT1*, *RMST*, *MEG3* and *MT1DP*)	Levels of *LUCAT1*, *RMST* and *MEG3* were lower in patients with BD, specifically in male patients with BD, compared with HCs
[37]	63 patients with BD, 42 with MDD and 55 HCs	PBMC	miRNA	qPCR	miR-499-5p	Higher levels of miR-499-5p in patients with BD (regardless of disease phase) compared with HCs
[22]	Serum: 41 patients with BD, 43 with MDD and 93 HCs; fibroblasts: 12 patients with BD, 23 with MDD and 15 HCs	Serum and fibroblasts	mRNA	qPCR	*IGFBP2*	Lower *IGFBP2* levels exclusively in patients with BD compared with HCs
[38]	37 rapid cycling patients with BD in different affective states and 40 HCs	PBMC	mRNA	qPCR	19 candidate genes	Lower levels of *POLG* and *OGG1* in patients with BD compared with HCs; higher levels of *NDUFV2* in patients in a depressed state compared with those in an euthymic state. A gene expression score including all genes showed an AUC of 0.73 in separating patients from HCs
[39]	50 patients with BD and 50 HCs	PBMC	lncRNA	qPCR	*DISC1* and *DISC2*	Lower levels of *DISC1* and higher levels of *DISC2* in patients with BD compared with HCs (AUC: 0.76 and 0.68, respectively)
[40]	50 patients with BD and 50 HCs	PBMC	lncRNA	qPCR	6 apoptosis-related lncRNAs (*CCAT2*, *TUG1*, *PANDA*, *NEAT1*, *FAS-AS1* and *OIP5-AS1*)	Levels of *CCAT2*, *TUG1* and *PANDA* were higher and levels of *OIP5-AS1* were lower in patients with BD patients compared with HCs. *CCAT2* and *TUG1* expression levels were only different in male subgroups
[23]	11 drug-free manic psychotic patients with BD and 9 HCs	Plasma	miRNA	Nanostring and qPCR	Genome wide	Higher levels of hsa-miR-25-3p and hsa-miR-144-3p, and lower levels of hsa-miR-6721-5p in patients with BD compared with HCs
[41]	66 patients with BD and 66 HCs	Plasma	miRNA	qPCR	15 miRNAs	A model including levels of miR-15b-5p, miR-155-5p, miR-134-5p and miR-652-3p showing a 83.3% sensitivity and 78.8% specificity
[42]	56 patients with BD I and 52 HCs	Whole blood	miRNA	qPCR	hsa-miR-145-5p, hsa-miR-376a-3p, hsa-miR-3680-5p, hsa-miR-4253-5p, hsa-miR-4482-3p and hsa-miR-4725	Higher levels of hsa-miR-376a-3p, hsa-miR-3680-5p, hsa-miR-4253-5p, hsa-miR-4482-3p and lower levels of hsa-miR-145-5p in patients with BD compared with HCs
[43]	50 patients with BD and 50 HCs	Whole blood	lncRNA	qPCR	Five NF-κB-associated lncRNAs (*ANRIL*, *CEBPA-DT*, *H19*, *NKILA* and *HNF1A-AS1*)	Lower levels of *ANRIL*, *CEBPA-DT* and *HNF1-AS1* and higher levels of *NKILA* in patients with BD compared with HC. *HFN1A-AS1* showed the best diagnostic parameters (AUC: 0.86).

ANRIL, CDKN2B antisense RNA 1; AUC, area under the curve; BD I, bipolar disorder type 1; BD II, bipolar disorder type II; CCAT2, colon-cancer-associated transcript 2; CEBPA-DT, CEBPA-divergent transcript; COMT, catechol-O-methyltransferase; CYP27B1, cytochrome P450 family 27 subfamily B member 1; DISC1, DISC1 scaffold protein; DISC2, DISC2 scaffold protein; DNMTs, DNA methyltransferases; GAD67, glutamate decarboxylase; EVs, extracellular vesicles; FAT-AS1, FAS antisense RNA 1; GCAT, glycine C-acetyltransferase; H19, H19-imprinted maternally expressed transcript; HNF1A-AS1, HNF1A antisense RNA 1; IGFBP2, insulin-like growth factor-binding protein 2; LUCAT1, lung cancer-associated transcript 1; MALAT1, metastasis-associated lung adenocarcinoma transcript 1; MDD, major depressive disorder; MECP2, methyl CpG-binding protein 2; MEG3, maternally expressed 3; MT1DP, metallothionein 1D, pseudogene; NDUFV2, NADH:Ubiquinone oxidoreductase core subunit V2; NGS, next-generation sequencing; NEAT1, nuclear paraspeckle assembly transcript 1; NMDAR, N-methyl-D-aspartate receptor; NKILA, NF-KappaB interacting LncRNA; NOTCH4, notch receptor 4; NRG1, neuregulin 1; OGG1, 8-oxoguanine DNA glycosylase; OIP5-AS1, OIP5 antisense RNA 1; PANDA, ATPase copper transporting beta; PBMC, peripheral blood mononuclear cells; PDYN, prodynorphin; POLG, DNA Polymerase Gamma, Catalytic Subunit; PSAT1, phosphoserine aminotransferase 1; qPCR, quantitative PCR; RMST, rhabdomyosarcoma 2-associated transcript; SERT, serotonin transporter; SHMT2, serine hydroxymethyltransferase 2; SNHG6, small nucleolar RNA host gene 6; SLC1A4, solute carrier family 1 member 4; SRR, serine racemase; TUG1, taurine upregulated 1; VDR, vitamin D receptor.

### 2.2. Levels of RNA Markers in Cellular Models Derived from Patients with BD

Studies investigating RNA biosignatures of BD in cellular models derived from patients with BD are shown in Table 2. A number of studies investigating RNA biosignatures of BD (or of response to mood stabilizers, as shown in Section 3.2) were conducted in lymphoblastoid cell lines (LCLs). While some studies did not identify significant differences in RNA markers between LCLs derived from patients with BD and HCs [44], a recent study including 37 euthymic patients with BD I and 20 HCs suggested a potential role for circadian genes, showing lower levels of aryl hydrocarbon receptor nuclear translocator-like protein 1 (ARNTL) and higher levels of circadian-associated repressor of transcription (CIART) and basic helix–loop–helix family member E41 (BHLHE41) in patients with BD compared with HCs [45]. Interestingly, genes related to the regulation of circadian rhythms was also implicated in a study conducted by the same authors in response to lithium in patients with cluster headache [46]. Other studies aimed at identifying biomarkers of specific endophenotypes of BD such as suicide risk. Squassina and colleagues investigated differences at baselines, as well as after in vitro lithium treatment in LCLs, from 9 patients with BD who died by suicide, 17 at low risk of suicide, 17 at high risk of suicide and 21 HCs [47]. In this study, in vitro treatment with lithium chloride (LiCl) 1 mM for 1 week, increased expression of the spermidine/spermine N1-acetyltransferase 1 (SAT1) gene in LCLs from HCs or from patients with BD at low or high risk of suicide but not in those from patients with BD who died by suicide. The enzyme encoded by this gene is a key regulator of cellular content of polyamines, a system of ubiquitous molecules involved in cell growth, differentiation and stress response, previously suggested to be altered in suicide [48]. In a subsequent study, the same group conducted a miRnome analysis showing higher levels of miR-4286 and lower levels of miR-186-5p in LCLs from patients who died by suicide compared with patients at low risk of suicide and HCs. Based on an in silico analysis, this study also suggested that a higher expression of miR-4286 might be responsible for a reduction in the expression of several genes involved in glucose metabolism [49]. The use of LCLs as a cellular model provides a number of advantages that contributes to their widespread use, such as the possibility to minimize variability by growing the cells under strictly similar conditions, as well as testing the effect of in vitro treatment. However, LCLs also present some criticisms that are still under debate, mainly concerning the effects of immortalization on the host genome. While LCLs are still used, over the last few years, cellular models for the identification of RNA biosignatures of BD shifted to NPCs, differentiated neurons or brain organoids derived from iPSCs. In some of these studies, cellular models were used to explore mechanistic aspects and corroborate hypotheses developed based on *post mortem* brain samples. Among these, Bavamian and colleagues conducted a candidate miRNA–mRNA study, investigating the levels of miR-34a and predicted targets [50]. The authors showed increased levels of this miRNA in *post mortem* cerebellum samples from 29 patients with BD and 34 HCs and subsequently explored the effect of the enhancement of miR-34a expression in iPSC-derived NPCs from 1 patient with BD and 1 HC. Increased expression of this miRNA was associated with impaired neuronal differentiation, expression of synaptic proteins and neuronal morphology [50]. A transcriptomic study with a relatively large sample size was conducted by Kathuria and colleagues and included brain organoids from eight patients with BD I and eights HCs [51]. This study reported the downregulation of pathways involved in cell adhesion, neurodevelopment and synaptic biology and the upregulation of genes involved in immune signaling in organoids from patients with BD compared with HCs. A network analysis conducted on differentially expressed genes showed as the central hub the neurocan (NCAN) gene, which was significantly downregulated in brain organoids from patients with BD. *NCAN* encodes a proteoglycan component of the neuronal extracellular matrix, which is involved in remodeling of neuronal tissue, neural adhesion and migration. Interestingly, this locus has been previously found to be implicated in BD by GWAS [2]. The results from this study support the promising power and utility of three-dimensional cellular models to investigate the molecular determinants of BD.

## 3. RNA Biomarkers and Response to Mood Stabilizers

### 3.1. Peripheral Levels of RNA Markers in Biofluids or Peripheral Cells from Patients with BD Characterized for Response to Mood Stabilizers

The majority of the studies investigating the association between RNA markers and response to mood stabilizers in biofluids or peripheral cells from patients with BD focused on treatment with lithium salts (Table 3), with only few studies including patients treated with other mood stabilizers [57,58]. Among these, the study of Pandey and colleagues was conducted in a pediatric population including 19 patients with BD treated with mood stabilizers for 8 weeks and had a candidate gene design. The authors showed a positive correlation between the change in *BDNF* levels and the change in the Young Mania Rating Scale (YMRS) score at 8 weeks [57]. Another study conducted by Rong and colleagues measured plasma levels of miR-134 based on previous evidence, suggesting a role for this miRNA in synaptic development [58]. This study included 21 manic patients with BD who were drug free at the first sampling. The patients were treated with a combination of antipsychotics and either lithium (*n* = 14), valproate (*n* = 6) or oxcarbazepine (*n* = 1). Clinical response was evaluated at 2 and 4 weeks with the Bech–Rafaelsen Mania Scale (BRMS) score. The authors reported a negative correlation between the severity of manic symptoms and the level of miR-134 at either baseline, 2 weeks or 4 weeks [58]. 

Promising evidence supports a potential role of genes related to apoptosis as markers of clinical response to lithium. In the Lithium Treatment-Moderate dose Use Study (LiTMUS), 60 patients with BD were randomized to optimized treatment vs. optimized treatment + lithium at a moderate dose (the dose was maintained at 600 mg for the first 8 weeks and then adjusted by the psychiatrist as needed) [59]. Optimized treatment consisted in at least one Food and Drug Administration-approved mood stabilizer other than lithium and followed the most recent recommendations of the Texas Implementation of Medication Algorithm. Peripheral blood genome-wide gene expression was measured at baseline and at 1 month after treatment initiation, while clinical response was assessed after 6 months with the Clinical Global Impression Scale for Bipolar Disorder–Severity (CGI-BP-S) score. The study found 62 genes to be differentially expressed after treatment (of which 18 were upregulated and 44 downregulated) exclusively in lithium responders. These changes were observed specifically in responders to lithium and not in responders to optimized therapy without lithium [59]. The BCL2-Like 1 (BCL2L1) gene showed the greatest difference between lithium responders and non-responders and was validated with quantitative PCR (qPCR). In addition, other negative regulators of apoptosis, such as phosphoinositide-3-kinase (PI3K) and mitogen-activated protein kinase 3 (MAP2K3), were found to be upregulated in lithium responders. These results confirm findings from a previous transcriptomic study in which whole blood RNA levels were measured in 20 patients with BD treated with lithium for 8 weeks and evaluated with the Hamilton Depression Rating Scale (HDRS) [60]. In this study, genes differentially expressed between lithium responders and non-responders were enriched for the regulation of apoptosis pathway. After 4 weeks, anti-apoptotic genes, such as BCL2 apoptosis regulator (BCL2) and insulin receptor substrate 2 (IRS2), were upregulated in responders and downregulated in non-responders, while pro-apoptotic genes, such as BCL2 antagonist/killer 1 (BAK1) and BCL2-associated agonist of cell death (BAD) were downregulated in responders and upregulated in non-responders, although these changes were not significant anymore at 8 weeks. Similarly, a study including 25 patients with BD during a major depressive episode, treated with lithium for 6 weeks and evaluated with the HDRS, showed that baseline *BCL2* levels predicted improvement of depressive symptoms after lithium therapy. In addition, the authors reported a significant association between changes in levels of *BCL2* and the change in the HDRS score after lithium treatment.

**Table 3:** Studies evaluating the association between RNA markers and response to mood stabilizers in biofluids or peripheral cells.

Ref.	Sample	RNA Source	RNA Type	Measurement Method	Targets	Main Findings
[61]	20 patients with BD (9 during a hypomanic episode, 11 during a depressive episode) treated with lithium for 8 weeks and 15 HCs. Response was evaluated with the HDRS, YMRS, CGI-I and CGI-S	Whole blood	mRNA	Microarray	Genome wide	13 genes showed a nominally significant change after treatment, and this change was correlated with a change in the clinical severity measured with the CGI-S score
[62]	21 patients treated with lithium for 8 weeks. Response was evaluated with HDRS, YMRS, CGI-I and CGI-S, 16 HCs	Whole blood	mRNA	Microarray	Genome wide	The sphingomyelin metabolism pathway was associated with the change in the HDRS score, and this effect was found to be mediated by the volume of the mediodorsal thalamus measured with brain MRI scans
[59]	60 patients treated with OPT or OPT + lithium moderate dose for 6 months. Response was evaluated with the CGI-BP-S	Whole blood	mRNA	Microarray and qPCR	Genome wide	In patients treated with lithium, 62 genes were differentially regulated in responders compared with non-responders, with *BCL2L1* showing the greatest difference
[63]	21 patients with BD treated with lithium and antipsychotics and 20 HCs. Response was evaluated with the YMRS	Whole blood	mRNA	qPCR	*TERT*	*TERT* levels were upregulated both at baseline and at remission in patients with BD compared with HCs
[60]	20 patients treated with lithium for 8 weeks and evaluated with the HDRS	Whole blood	mRNA	Microarray	Genome wide	Genes differentially expressed between responders and non-responders were enriched for the regulation of apoptosis pathway. After 4 weeks, anti-apoptotic genes such as *BCL2* and *IRS2* were upregulated in responders and downregulated in non-responders, while pro-apoptotic genes such as *BAK1* and *BAD* were downregulated in responders and upregulated in non-responders. These changes were not significant anymore at 8 weeks
[64]	25 patients with BD during a major depressive episode, treated with lithium for 6 weeks, 31 HCs. Response was evaluated with the HDRS	Whole blood	mRNA	qPCR	5 genes part of the AKT/mTOR pathway	Significant association between changes in levels of *AKT-1*, *BCL2* and changes in the HDRS score after lithium treatment; baseline *BCL2* levels predicted improvement of depressive symptoms after lithium therapy
[57]	19 pediatric patients with BD treated with mood stabilizers for 8 weeks and evaluated with the YMRS	Whole blood	mRNA	qPCR	*BDNF*	Positive correlation between the change in *BDNF* levels and the change in the YMRS score at 8 weeks
[58]	21 patients with BD, drug-free at the first sampling. Response to treatment with antipsychotics and mood stabilizers was evaluated with the BRMS at 2 and 4 weeks	Plasma	mRNA	qPCR	miR-134	Negative correlation between miR-134 levels at baseline, 2-week or 4-week follow-up and severity of manic symptoms
[65]	50 patients with long-term lithium treatment and evaluated with the Alda scale	Whole blood	mRNA	NGS	Genome wide	Nominal association between lithium response and a co-expression module (the central modulators of which were mitochondrially encoded genes). A total of 43 out of the 46 genes included in this module showed reduced levels in responders compared with non-responders

AKT1, AKT serine/threonine kinase 1; BAD, BCL2-associated agonist of cell death; BAK1, BCL2 antagonist/killer 1; BCL2, BCL2 apoptosis regulator; BCL2L1, BCL2-like 1; BD, bipolar disorder; BDNF, brain-derived neurotrophic factor; BRMS, Bech–Rafaelsen Mania Scale; CGI-I, Clinical Global Impression Scale Improvement; CGI-BP-S, Clinical Global Impression Scale for Bipolar Disorder Severity; CGI-S Clinical Global Impression Scale Severity; GFAP, glial fibrillary acidic protein; HCs, healthy controls; HDRS, Hamilton Depression Rating Scale; IRS2, insulin receptor substrate 2; mRNA, messenger RNA; MRI, magnetic resonance imaging; qPCR, quantitative PCR; NGS, next-generation sequencing; TERT, telomerase reverse transcriptase; YMRS, Young Mania Rating Scale.

### 3.2. Levels of RNA Markers in Cellular Models Derived from Patients with BD Characterized for Response to Mood Stabilizers

All studies investigating differences in RNA levels between patient responders and non-responders to mood stabilizers using cellular models were focused on lithium, with the large majority being conducted using LCLs and only a few studies using neurons differentiated from iPSCs (Table 4). Most available studies investigated genome-wide levels of mRNAs or candidate genes, with only three studies integrating genome-wide levels of mRNAs and miRNAs [66,67,68]. Long-term lithium response was generally evaluated with the Alda scale [4,69], although some studies used different criteria, such as the rate of relapse [70] or evaluated short-term response to lithium treatment [71]. Among studies with a candidate gene design, promising results have been reported for genes playing a role in the regulation of circadian rhythms [72,73]. This pathway has been investigated based on a large body of evidence suggesting that disruption of circadian rhythms and persistent circadian/sleep alterations characterized patients with BD (even during the euthymic state) and that lithium is able to affect circadian rhythms in both animal models and in humans [72]. Based on the available studies, lithium has been suggested to be able to modulate the expression of circadian genes with differences in amplitude and temporal evolution according to the patient’s lithium clinical response status.

Some studies that investigated both differences at baseline among responders and non-responders, as well as the effect of in vitro treatment with LiCl 1 mM for 1 week on gene expression, found the largest differences to be based on clinical response rather than on the effect of in vitro treatment [68,74]. Among these, there is a recent study from Niemsiri and colleagues, which included prox1+ hippocampal dentate gyrus (DG)-like neurons derived from six patients responders to lithium, five non-responders and six HCs. This study reported 41 genes to be differentially expressed between responders and non-responders, regardless of in vitro treatment. A functional enrichment analysis suggested focal adhesion and the extracellular matrix to be the most significantly enriched functions [74]. Nonetheless, a previous study conducted using the same cellular model showed in vitro treatment with LiCl 1 mM for 1 week to significantly modulate 560 genes in responders and 40 genes in non-responders. Genes for which lithium rescued expression in responders were related to the PKA/PKC pathways, action potential firing and mitochondria [75]. This landmark study also showed that neurons derived from patients with BD display a hyperexcitability phenotype that was normalized by lithium treatment exclusively in neurons derived from patients who had a clinical history of response to this drug [75]. Evidence from another transcriptomic study conducted in DG neurons suggested the hyperexcitability phenotype to be possibly related to alterations of the Wnt/β–catenin signaling pathway and decreased levels of the lymphoid enhancer-Binding factor 1 (LEF1) transcription factor, which were observed in neurons derived from lithium non-responders [76].

**Table 4:** Studies evaluating the association between RNA markers and response to mood stabilizers in cellular models.

Ref.	Sample	RNA Source	RNA Type	Measurement Method	Targets	Main Findings
[70]	16 patients treated with lithium. Response was evaluated based on the rate of relapse	LCLs	mRNA	NGS	Genome wide	In vitro treatment with LiCl 1 mM for 1 week modulated 22 coexpression modules
[66]	20 patients with BD (11 responders and 9 non-responders). Response was evaluated with the Alda scale	LCLs	mRNA, miRNA	Microarray	Genome wide	335 genes (217 upregulated and 118 downregulated) and 77 miRNAs (46 upregulated and 31 downregulated) were nominally differentially expressed between responders and non-responders
[72]	36 patients with long-term lithium treatment. Response was evaluated with the Alda scale	LCLs	mRNA	qPCR	20 circadian genes	Differential temporal evolution between non-responders and responders for levels of different circadian genes
[67]	16 patients with long-term lithium treatment. Response was evaluated with the Alda scale	LCLs	mRNA, miRNA	Microarray, qPCR	Genome wide	In vitro treatment with LiCl 1 mM for 1 week induced downregulation of *THRAP3* and *TFAM* in responders
[77]	8 patients with BD with long-term lithium treatment. Response was evaluated with the Alda scale	LCLs, fibroblasts	mRNA	Microarray, qPCR	Genome wide	No significant difference in gene expression levels based on lithium response
[71]	22 patients treated with lithium for 6 weeks. Response was evaluated with the MADRS and YMRS at 6 weeks	Olfactory neurons	mRNA	qPCR	*GSK3B, AKT1, PRKCE* and *CRMP1*	Treatment-associated downregulation of *CRMP1* predicted improvement of both manic and depressive symptoms
[75]	6 patients treated with lithium. Response was evaluated with the CGI at 4 months	Neurons differentiated from iPSCs	mRNA	NGS	Genome wide	In vitro treatment with LiCl 1 mM for 1 week modulated 560 genes in responders and 40 genes in non-responders. Genes for which lithium rescued expression in responders were related to the PKA/PKC pathways, action potential firing and mitochondria
[78]	17 patients with long-term lithium treatment. Response was evaluated with the Alda scale	LCLs	miRNA	qPCR	let7-c	Nonsignificant trend for higher let-7c expression in non-responders compared with responders
[79]	24, 41 and 17 patients with long-term lithium treatment. Response was evaluated with the Alda scale	LCLs	mRNA	NGS, qPCR	Genome wide	56 genes showed nominal differential expression between responders and non-responders. *HDGFRP3* and *ID2* were validated in the independent cohorts
[80]	36 patients with BD with long-term lithium treatment. Response was evaluated with the Alda scale	LCLs	mRNA	qPCR	*GADL1*	No difference in *GADL1* levels between responders and non-responders. In vitro treatment with LiCl 1 mM for 4 or 8 days did not modify *GADL1* levels
[74]	6 patients with BD responders to lithium, 5 patients with BD non-responders to lithium and 6 HCs. Response was evaluated with the Alda scale	Neurons differentiated from iPSCs	mRNA	NGS, qPCR	Genome wide	41 genes were differentially expressed between responders and non-responders, regardless of in vitro treatment with LiCl 1 mM for 1 week. Focal adhesion and the extracellular matrix were the most significant functions based on functional enrichment analysis of the top 500 proximal network genes
[81]	20 patients with long-term lithium treatment. Response was evaluated with the Alda scale	LCLs	mRNA	Microarray, qPCR	*RBM3*	*RBM3* was upregulated in responders compared with non-responders. In vitro treatment with LiCl 1 mM for 1 week did not modify *RBM3* levels
[82]	LCL: 25 patients with long-term lithium treatment and 12 HCs. NPC: 2 patients with BD. Response was evaluated with the Alda scale	LCLs, NPCs	mRNA	NGS, qPCR	*BCL2, GSK3B* and *NR1D1*	In vitro treatment with LiCl 1 mm for 1 week increased the expression of *BCL2* and *GSK3B* in responders
[83]	20 patients with long-term lithium treatment. Response was evaluated with the Alda scale	LCLs	mRNA	Microarray, qPCR	Genome wide	In vitro treatment with LiCl 1 mm for 1 week modified levels of 29 genes, including *ZNF493* and *ZNF429*, in responders
[73]	20 patients with long-term lithium treatment. Response was evaluated with the Alda scale	LCLs	mRNA	Microarray	17 circadian genes	Higher levels of *BHLHE40* in responders compared with non-responders
[68]	20 patients with long-term lithium treatment. Response was evaluated with the Alda scale	LCLs	miRNA, mRNA	Microarray, NGS, qPCR	Genome wide	miR-320a, miR-155-3p and three of their targeted genes (*CAPNS1* and *RGS16* for miR-320 and *SP4* for miR-155-3p) were differentially expressed between responders and non-responders
[76]	3 patients with BD responders to lithium, 3 non-responders and 4 HCs. Response was evaluated with the Alda scale	Neurons differentiated from iPSCs	mRNA	NGS, qPCR	Genome wide	Alterations of the Wnt/β-catenin signaling pathway and decreased levels of the *LEF1* transcription factor, which were observed in neurons derived from lithium non-responders
[84]	30 patients with long-term lithium treatment. Response was evaluated with the Alda scale	LCLs	mRNA	qPCR	*PDLIM5*	No association between *PDLIM5* levels and response
[85]	20 and 12 patients with long-term lithium treatment. Response was evaluated with the Alda scale	LCLs	mRNA	Microarray, qPCR	Genome wide	2060 genes were differentially expressed between responders and non-responders; *IGF1* was validated in the independent sample
[86]	12 patients (all responders to long-term lithium treatment according to [87])	LCLs	mRNA	Microarray, Northern blot	Genome wide	In vitro treatment with LiCl 1 mM for 1 week decreased the expression of 7 genes

AKT1, AKT serine/threonine kinase 1; BCL2, BCL2 apoptosis regulator; BDNF, brain-derived neurotrophic factor; BHLHE40, basic helix–loop–helix family member E40; CAPNS1, calpain small subunit 1; CRMP1, collapsin response mediator protein 1; GADL1, glutamate decarboxylase-like 1; GO, gene ontology; GSK3B, glycogen synthase kinase 3 beta; HDGFRP3, hepatoma-derived growth factor, related protein 3, isoform CRA_a; ID2, inhibitor of DNA-binding 2; IGF1, insulin-like growth factor 1; LCL, lymphoblastoid cell line; LEF1, lymphoid enhancer-binding factor 1; LiCL, lithium chloride; mRNA, messenger RNA; miRNA, microRNA; NR1D1, nuclear receptor subfamily 1 group D member 1; PDLIM5, PDZ and LIM domain 5; PKA A, protein kinase A; PKC, protein kinase C; PRKCE, protein kinase C epsilon; RBM3, RNA-binding motif protein 3; RGS16, regulator of G protein signaling 16; SP4, Sp4 transcription factor; TFAM, transcription factor A mitochondrial; THRAP3, thyroid hormone receptor-associated protein 3; ZNF429, zinc finger protein 493; ZNF 429, zinc finger protein 493.

## 4. Conclusions

Over the last few years, a growing body of research has contributed to promising findings of the potential role of coding and noncoding RNAs in the pathogenesis of BD and in response to mood stabilizers. While the majority of the first studies were aimed at discriminating patients with BD from nonpsychiatric controls, another relevant but less explored area is related to the discrimination of patients with BD from patients with MDD. Indeed, the differential diagnosis between BD and MDD is challenging and represents a major clinical problem, especially in the first phases of the disease. Nonetheless, it has relevant prognostic and therapeutic implications, since the misdiagnosis or mistreatment of BD has been associated with a more severe disease course and a higher number of hospitalizations and suicide attempts [88], as well as increased costs [89]. While still limited in number and in sample size [20,29], studies focused on this topic provide promising preliminary findings and might help to distinguish trans-disorder biosignatures from those specific for different psychiatric disorders.

A promising approach applied in a growing number of studies is the determination of the transcriptomic profiling of plasma or serum exosome miRNAs. Exosomes carry bioactive molecules to adjacent or distant cells, thus playing a vital role in intercellular communication and signal transmission. Neural exosomes can cross the blood–brain barrier, thus potentially allowing to identify brain-relevant biosignatures of disease and drug response in a noninvasive way. Based on this premises, the change in the peripheral exosome content in patients with BD according to disease course or treatment response might allow to measure easily accessible biomarkers capable of, at least, partly reflecting cellular and molecular events ongoing in the brain. While these studies are still in their infancy, promising evidence has been reported regarding the ability of exosome miRNA biosignatures to distinguish between patients with BD and HCs, while no significant differences have been reported among different states of BD [25].

Finally, studies investigating the role of transcripts as biomarkers of BD and/or clinical response to mood stabilizers are now taking advantage of novel cellular models such as NPCs and neurons derived from iPSCs from patients characterized for lithium response, as well as tridimensional models such as brain organoids. By allowing to study the function of living human neurons carrying the genetic background of patients with a specific phenotype, as well as to test the effect of in vitro treatment with drugs, these models have the potential to address some of the shortcomings of previous cellular models and bring us closer to precision medicine in BD.

## Figures and Tables

**Table 2:** Studies evaluating the association between RNA markers and bipolar disorder in cellular models.

Ref.	Sample	RNA Source	RNA Type	Measurement Method	Targets	Main Findings
[52]	3 patients with BD and 3 HCs	Neurons differentiated from iPSC-derived NPCs	mRNA, miRNA	Microarray, qPCR	Genome wide	Six (miR-128-3p, miR-138-2-3p, miR-195-5p, miR-382-5p, miR-487b-3p and miR-744-3p) and two miRNAs (miR-10b-5p and miR-10b-3p) showed higher or lower levels, respectively, in neurons derived from patients with BD compared with HCs
[50]	iPSC-derived NPCs from 1 patient with BD and 1 HC; post-mortem brain samples from 29 patients with BD and 34 HCs	NPCs, post-mortem brain samples	mRNA, miRNA	Nanostring, qPCR	miR-34a and predicted targets	Increased miR-34a levels in the cerebellum of patients with BD compared with HCs. In NPCs, the enhancement of miR-34a expression impaired neuronal differentiation, expression of synaptic proteins and neuronal morphology
[45]	37 euthymic patients with BD I and 20 HCs	LCLs	mRNA	NGS, qPCR	19 circadian genes	Lower levels of *ARNTL* and higher levels of *CIART* and *BHLHE41* in patients with BD compared with HCs
[44]	62 patients with BD I and 17 HCs	LCLs	mRNA	Microarray, qPCR	Genome wide	No significant difference between patients with BD and HCs
[53]	iPSC and NPCs from 6 patients with BD and 4 HCs; post-mortem brain samples from 35 patients with BD and 34 HCs (BA46), 15 patients with BD and 15 HCs (corpus callosum and BA8)	NPCs, iPSCs and post-mortem brain samples	mRNA, lncRNA	qPCR	*BDNF* and *BDNF-AS*	*BDNF* expression was lower in iPSCs but higher in NPCs from BD patients compared with HCs. BDNF expression was lower in BA46 but not in BA8 or corpus callosum from patients with BD compared with HCs
[51]	8 patients with BD I and 8 HCs	Brain organoids generated from iPSCs	mRNA	NGS	Genome wide	Downregulation of pathways involved in cell adhesion, neurodevelopment and synaptic biology and upregulation of genes involved in immune signaling in organoids from patients with BD compared with HCs. The central hub in the network analysis was the neurocan gene, located in a locus with evidence for genome-wide significant association for BD
[54]	4 patients with BD and 4 HCs	Neurons and NPCs derived from iPSCs	mRNA	Microarray, qPCR	Genome wide	328 genes were differentially expressed neurons from patients with BD and HCs. These genes were enriched for alterations in RNA biosynthesis and metabolism, protein trafficking and receptor signaling pathways. Higher levels of GAD1 in neurons from patients with BD were confirmed with qPCR
[55]	2 brothers with BD and 2 unaffected parents	NPCs derived from iPSCs	mRNA	Nanostring, NGS	Genome wide	NPCs expressing CXCR4 from both BD patients compared to their unaffected parents showed differences in the expression of genes critical for neuroplasticity, including Wnt pathway components and ion channel subunits
[56]	2 monozygotic twins discordant for schizoaffective disorder, bipolar type, and 2 pairs of monozygotic twins discordant for SZ	Brain organoids generated from iPSCs	mRNA	scRNAseq	Genome wide	Enhanced GABAergic specification and reduced cell proliferation following diminished Wnt signaling in the patient with BD, which was confirmed in iPSC-derived forebrain neuronal cells
[47]	9 patients with BD who died by suicide, 17 at low risk of suicide, 17 at high risk of suicide and 21 HCs	LCLs	mRNA	qPCR	*SAT1*	In vitro treatment with LiCl 1 mM increased *SAT1* expression in the high and low risk groups as well as in HCs, but not in suicide completers
[49]	7 patients with BD who died by suicide, 11 patients at low risk of suicide and 12 HCs	LCLs, NPCs and post-mortem brain samples	miRNA	Nanostring, qPCR	Genome wide	Higher levels of miR-4286 and lower levels of miR-186-5p in LCLs from patients who died by suicide compared with patients at low risk of suicide and HCs. In vitro treatment with lithium reduced miR-4286 expression in human NPCs

ARNTL1, basic helix–loop–helix ARNT-like 1; BD I, bipolar disorder type 1; BDNF, brain-derived neurotrophic factor; BHLHE41, basic helix–loop–helix family member E41; CIART, circadian-associated repressor of transcription; CXCR4, CXC chemokine receptor-4; GAD1, glutamate decarboxylase; HCs, healthy controls; iPSCs, induced pluripotent stem cells; lncRNA, long noncoding RNA; LCLs, lymphoblastoid cell lines; mRNA, messenger RNA; microRNA, miRNA; NGS, next-generation sequencing; NPCs, neural precursor cells; qPCR, quantitative PCR; SAT1, spermidine/spermine N1-acetyltransferase; scRNAseq, single cell RNA sequencing.

## Data Availability

Not applicable.
